# Applying a statistical method in transvaginal ultrasound training: lessons from the learning curve cumulative summation test (LC-CUSUM) for endometriosis mapping

**DOI:** 10.1186/s10397-017-1022-4

**Published:** 2017-10-03

**Authors:** Vered H. Eisenberg, Juan L. Alcazar, Nissim Arbib, Eyal Schiff, Reuven Achiron, Motti Goldenberg, David Soriano

**Affiliations:** 1 Department of Obstetrics and Gynecology, Sheba Medical Center, Tel-Hashomer, Tel-Aviv University, 52621 Ramat Gan, Israel; 2 Department of Obstetrics and Gynecology, Clinica Universidad de Navarra, University of Navarra, Pamplona, Spain

**Keywords:** Transvaginal ultrasound, Endometriomas, Deep infiltrative endometriosis, Learning curve, LC-CUSUM, Individualized assessment

## Abstract

**Background:**

Methods available for assessing the learning curve, such as a predefined number of procedures or direct mentoring are lacking. Our aim was to describe the use of a statistical method to identify the minimal training length of an experienced sonographer, newly trained in deep infiltrating endometriosis (DIE) mapping by evaluating the learning curve of transvaginal ultrasound (TVUS) in the preoperative assessment of endometriosis.

**Methods:**

A retrospective study in a tertiary referral center for endometriosis. Reports and stored data from TVUS scans performed by one operator with training in general gynecological ultrasound, but not in endometriosis mapping, were analyzed retrospectively for patients who subsequently underwent laparoscopy, which served as a reference standard. The performance of TVUS was assessed for the following sites: endometriomas, bladder, vagina, pouch of Douglas, bowel and uterosacral ligaments, and correlated with laparoscopic findings. Sensitivity, specificity, PPV, NPV, and accuracy were calculated, and the operator’s diagnostic performance was assessed using the learning curve cumulative summation test (LC-CUSUM).

**Results:**

Data from 94 women were available for analysis. The learning curve using the LC-CUSUM graph showed that the sonographer reached the predefined level of proficiency in detecting endometriosis lesions after 20, 26, 32, 31, 38, and 44 examinations for endometriomas, bladder nodules, vaginal nodules, pouch of Douglas obliteration, bowel nodules, and uterosacral ligament nodules, respectively.

**Conclusions:**

LC-CUSUM allows monitoring of individual performance during the learning process of new methodologies. This study shows that a sonographer trained in general gynecologic ultrasonography, who devotes time to learn TVUS for DIE mapping, can achieve proficiency for diagnosing the major types of endometriotic lesions after examining less than 50 patients who subsequently undergo surgery in a training setting.

## Background

Endometriosis is a common benign gynecological condition with a prevalence rate of up to 15% [1] in reproductive age women. It is defined as the presence of endometrial tissue, glands and stroma outside the endometrial cavity. The clinical manifestations of endometriosis vary widely and may include secondary dysmenorrhea, chronic pelvic pain, dyspareunia, dyschezia, intermittent diarrhea and constipation, hematochezia, dysuria, pain on urination, irritable bladder, and hematuria. The mean age at diagnosis of endometriosis is 25–29 years and may be higher in women who present with infertility rather than pelvic pain [2]. Deep infiltrating endometriosis (DIE) is defined as endometriotic lesions that penetrate more than 5 mm from under the peritoneum [1]. These may be multifocal and are most commonly found in the uterosacral ligaments, posterior vaginal fornix, retro cervical region, rectovaginal septum, vesicouterine pouch, bladder, and anterior wall of the recto sigmoid.

The available pre-operative diagnostic tools include the patient’s history, pelvic examination, transvaginal ultrasonography (TVUS), and magnetic resonance imaging (MRI) [3, 4]. A negative imaging workup does not exclude endometriosis. An average diagnostic delay from symptom onset till definitive diagnosis can be up to 12 years [5, 6]. Chronic pelvic pain and infertility significantly affect the patient’s quality of life and carry a high economic burden [7]. It is imperative that a prompt and accurate diagnosis is reached in order to bypass the diagnostic delay and refer patients for adequate care by endometriosis specialists in dedicated centers [8], to improve counseling and preoperative preparation and to determine the multidisciplinary team to be present in order to allow definitive surgical treatment.

While it is recognized that TVS should be the first-line imaging examination for the preoperative work-up of patients [9], its performance for diagnosing endometriosis and more specifically endometriomas, has been shown to be accurate only in the hands of experienced sonographers [3, 10–14]. Most experienced sonographers will easily identify endometriotic cysts [4, 12, 13], whereas sonographic diagnosis of DIE lesions requires skill and dedication, after which reported accuracy can be as high as 99% [3, 4, 8–11, 14]. The determination of how much experience is required to reach proficiency in the diagnosis of DIE is extremely important for planning training programs, and is not sufficiently known [4, 8].

Biau et al. introduced the learning curve cumulative summation test (LC-CUSUM) that was specifically designed to determine when a level of proficiency has been reached. The LC-CUSUM is a statistical tool which is performed in order to indicate when a process has reached a predefined level of performance [15–17]. The importance of establishing a learning curve is significant for quality assurance, patient safety, and cost analysis, and has been previously described for varied procedures, in surgery and in obstetrics and gynecology.

The purpose of this study was to determine the learning curve of TVUS in the preoperative assessment of endometriosis and in particular DIE by an experienced sonographer, newly trained in endometriosis mapping.

## Methods

This study evaluated the performance of dedicated TVUS for the diagnosis of endometriosis and DIE. All the examinations were performed by the same sonographer with previous experience in gynecological ultrasound but without previous experience in DIE mapping expect for an introductory course. She had previously performed more than 10,000 gynecological general examinations over the years but had not performed dedicated endometriosis scanning apart from the incidental endometriomas and no specific DIE scanning. She had undergone training and learning DIE mapping as described by previous authors [3, 8–11, 14] during 2010–2011. Starting from May 2011, all patients referred to our endometriosis center underwent TVS as preoperative assessment. Out of 250 patients who were examined during the study period, the 94 who underwent surgery at our institution were included in the analysis. The remaining women either did not qualify for surgery or were operated on at another institution. The indications for surgery were intractable pain or infertility. The patient’s clinical history and symptoms were obtained from the electronic hospital records. These data included patient’s age, body mass index (BMI, kg/m^2^), parity, previous cesarean sections, smoking history, dysmenorrhea, dyspareunia, urinary and gastrointestinal symptoms, infertility history, fertility treatment and type, and number of previous IVF cycles.

The TVUS were carried out using a transvaginal 5–9 MHz probe with 2D/3D capabilities (Voluson 730 or E6, GE Medical Systems). Images were interpreted in real-time and were stored for later analysis. The examination was performed in a standardized way for all patients. This included a thorough evaluation of all pelvic viscerae, for lesions consistent with endometriosis: endometriomas, tubal adhesions, vagina, posterior and lateral vaginal fornices, the retro cervical area with torus uterinum, the parametria laterally, the rectovaginal septum, the bowel, peritoneal surfaces, bladder and vesicouterine pouch, and uterosacral ligaments, as previously described by other authors [18–20]. Organ mobility was evaluated in the anterior and posterior compartment. Movement of the posterior surface of the uterus, cervix or vagina in relation to the bowel was examined in order to determine pouch of Douglas obliteration, previously described as the “sliding sign” [21, 22]. For a more thorough description of the methodology, please refer to the Appendix.

The reference standard for diagnosis was defined as surgical findings during laparoscopy. Success of the ultrasound procedure was defined as agreement between TVUS findings and surgical findings. All of the patients underwent laparoscopic surgery by trained endoscopic surgeons in a multidisciplinary team which included also urological and colorectal surgeons as required. Pelvic endometriosis was diagnosed based on any of the following: presence of endometrial tissue (endometrial glands and stroma) in pathological examination in at least one resected lesion, direct visualization of deep pelvic lesions of endometriosis associated with only fibrosis at biopsy, direct visualization of deep pelvic lesions of endometriosis which could not be resected, or complete cul-de-sac obliteration secondary to endometriosis which was deemed unresectable, because of a significant surgical risk to adjacent structures. The severity of endometriosis at surgery was evaluated retrospectively by one of the co-authors who were blinded to the ultrasound report, based on the Revised American Society for Reproductive Medicine (ASRM) Classification [23] and the histo-pathological reports were reviewed. The findings at TVUS were compared with the descriptive visual findings at surgery. Pathological confirmation of the presence of endometriosis in resected lesions was obtained later but was not a prerequisite for inclusion as surgical findings are those that determine severity.

### Statistical analysis

Statistical analysis was performed using SPSS software (SPSS, IBM Corporation, Chicago, IL, USA). Continuous variables were expressed as means ± SD or medians, while categorical variables were expressed as frequencies and percentages. Sensitivity, specificity, positive predictive value (PPV), negative predictive value (NPV), and accuracy were calculated for the diagnosis of endometriomas, bladder nodules, vaginal nodules, pouch of Douglas obliteration, bowel nodules (including rectum, sigma, and Douglas pouch), and uterosacral ligament nodules with the results of laparoscopic surgery as the reference standard for diagnosis. The analysis was performed by anatomic location. Statistical significance was set at *P* < 0.05.

A standard CUSUM test monitors a sequential procedure with ability to reject the null hypothesis H_0_ that the process is in control [15], while the alternative hypothesis H_1_ is that the process is out of control. The process is deemed acceptable as long as the CUSUM score remains below a limit known as *h*. The learning curve summation (LC-CUSUM) test was used to assess whether the process has reached a predefined level of performance by signaling when the process can be considered to be in control [16, 17]. The LC-CUSUM sequentially tests the inverted hypotheses, the null hypothesis, namely, inadequate performance (H_0_), against the alternative hypothesis, namely, adequate performance (H_1_). It computes a score, from the successive outcomes, with successes yielding an increase in the score and failures yielding a decrease in the score, i.e., negative scores for correct interventions and positive scores for incorrect results are calculated as previously reported by Biau et al. [16, 17]. Once the summation score reaches a predefined level (h), the test rejects the null hypothesis in favor of the alternative hypothesis, which indicates an adequate performance level. The LU-CUSUM remains responsive at all times, so that as performance improves the trainee do not need to compensate unnecessarily for previous failures. Acceptable (*P*_1_) and unacceptable (*P*_0_) failure rates, the required level of performance and the properties of the test have to be set. Acceptable and unacceptable failure rates for this study were set at 10% (*P*_1_ = 0.10) and 25% (*P*_0_ = 0.25), respectively. These limits were chosen assuming that the pooled failure rates for an expert examiner could be around 10–25%, taking into account both false positive and false negative results. The failure rates were chosen based on the accuracy of TVUS for diagnosing endometriomas and DIE in experienced hands [24–26]. Type I (α) and type II (β) error rates were set at 0.1. A limit *h* = 2.0 was chosen from computer simulations which means that the risk of declaring a trainee proficient when his or her performance is inadequate was limited to 10% over 100 procedures. CUSUM values are plotted on the *y*-axis, and the number of examinations is plotted on the *x*-axis. Horizontal lines are plotted at regular intervals on the *y*-axis, defining *h0* and *h1* for the spacing between acceptable and unacceptable boundary lines, respectively. Competence is declared when the plot falls below two consecutive boundary lines.

Ethical approval was given from our local research ethics committee. Informed consent was not required as the ultrasound assessment was offered as part of standard clinical care at our center.

## Results

Ninety-four women were included in the analysis, all of whom underwent TVUS and subsequent laparoscopic surgery over the study period. Demographic data and patient symptoms are presented in Table 1. The median disease severity (ASRM) score at surgery was 43 (range 1–148), and the median ASRM stage was 4 (range 1–4): 15 (16%) patients had stage I, 4 (4.3%) stage II, 19 (20.2%) stage III and 56 (59.6%) had stage IV disease. All of the patients described long standing symptoms before being referred to our center. 49 (52%) women had had previous surgery for endometriosis.

**Table 1:** Demographic data and symptoms in 94 patients who underwent transvaginal sonography (TVUS) and subsequent laparoscopic surgery for endometriosis

Variable	Value (*n* = 94)
Age, mean ± SD, years (range)	34.1 ± 6.0 (20–47)
BMI, mean ± SD, kg/m^2^ (range)	23.6 ± 4.8 (16.9–40.2)
Parity, median (range)	0 (0–6)
Previous cesarean section (%)	12 (12.8)
Smoker (%)	28 (29.8)
Dysmenorrhea (%)	87 (92.6)
Dyspareunia (%)	60 (63.8)
Urinary complaints (%)	27 (28.7)
Gastrointestinal complaints (%)	51 (55.3)
Infertility (%)	34 (36.1)
Previous IVF treatments (%)	24 (25.5)
Number of IVF cycles, median (range)	5 (0–16)

Based on the gold standard of findings during surgery, 57 (60.6%) of women had endometriomas, 11 (11.7%) had bladder nodules, 39 (41.5%) had vaginal nodules, 48 (51.1%) had pouch of Douglas obliteration, 20 (21.3%) had bowel nodules (rectum, bowel, and pouch of Douglas), and 50 (53.2%) had uterosacral ligament involvement. Surgical findings in the 94 patients with pelvic endometriosis along with the sensitivity, specificity, PPV, NPV, and accuracy for TVUS findings and agreement with endometriosis findings at laparoscopy are presented in Table 2.

**Table 2:** Surgical findings in 94 patients with pelvic endometriosis. Sensitivity, specificity, PPV, NPV, and accuracy for TVUS findings and agreement with endometriosis findings at laparoscopy

Disease location	Cases (*n* = 94)	Sensitivity (%)	Specificity (%)	PPV (%)	NPV (%)	Accuracy (%)
Endometriomas	57 (60.6%)	100	100	100	100	100
Bladder nodules	11 (11.7%)	90.9	100	100	98.8	98.9
Vaginal nodules	39 (41.5%)	92.3	98.2	97.3	94.7	95.7
Pouch of Douglas obliteration	48 (51.1%)	93.8	91.3	91.8	93.3	92.5
Bowel lesions (rectum, sigma, POD)	20 (21.3%)	80	98.6	94.1	94.8	94.7
Uterosacral ligaments	50 (53.2%)	60	70.5	69.8	60.8	64.9

*PPV* positive predictive value, *NPV* negative predictive value, *TVUS* transvaginal ultrasound

### LC-CUSUM analysis

**Fig. 1:**
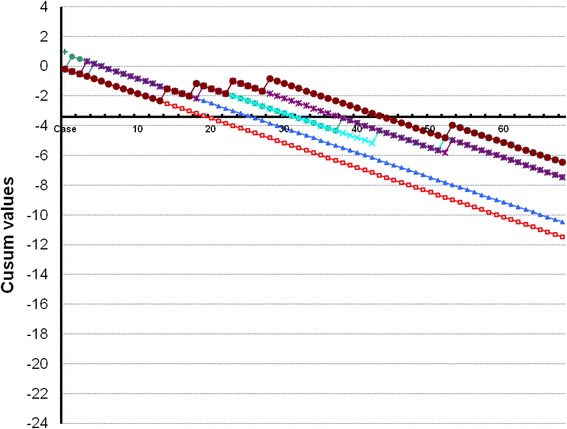
Cumulative summation test for the learning curve (LC-CUSUM) graphs for TVUS for endometriomas and deep infiltrative endometriosis. The vertical axis shows the CUSUM values, the horizontal axis shows the case number. Dotted horizontal lines show acceptable/unacceptable boundary lines of the CUSUM score. As long as the score remains over the limit *h* (dotted line), the operator is not considered as proficient, whereas when the LC-CUSUM score crosses this limit, he is considered to have become proficient. As long as the score remains under the limit, the operator is considered to maintain an acceptable performance. Performance was reached after 20 exams for endometriomas (red line), 26 exams for bladder nodules (blue), 32 exams for vaginal nodules (green), 31 exams for pouch of Douglas obliteration (turquoise), 38 exams for bowel nodules (purple), and 44 exams for uterosacral ligament nodules (dark red)

The cumulative summation test for the learning curve (LC-CUSUM) graphs for TVUS for endometriomas and DIE in our study is presented in Fig. 1.

#### Endometriomas

There were 57 endometriomas at surgery. The sonographer diagnosed all of the endometriomas correctly. There were also other lesions including one borderline serous tumor, one peritoneal cyst, one corpus luteum cyst, and one multicystic benign mesothelioma with decidual changes. None of these lesions was mistaken for an endometrioma, and they were all recognized as other lesions. Based on the LU-CUSUM curve it, takes the sonographer 20 examinations to become proficient at diagnosing endometriomas correctly.

#### Bladder lesions

**Fig. 2:**
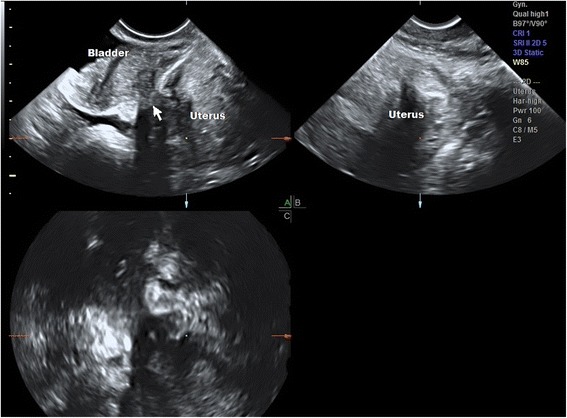
Multiplanar 3D image of TVUS of bladder detrusor endometriosis penetrating from the anterior uterine wall. See hourglass appearance of nodule penetration (arrow). The uterus is affected by adenomyosis

Eleven patients had bladder lesions at laparoscopy with penetration of the bladder wall and detrusor involvement (Fig. 2). The accuracy for their diagnosis was 99%. Based on the LU-CUSUM curve, it takes 26 examinations to become proficient at diagnosing bladder nodules correctly. There was one failed diagnosis by the sonographer in the fifth case from study start, the lesion was very small and was not resected by the surgeon due to lack of symptoms.

#### Vaginal lesions

**Fig. 3:**
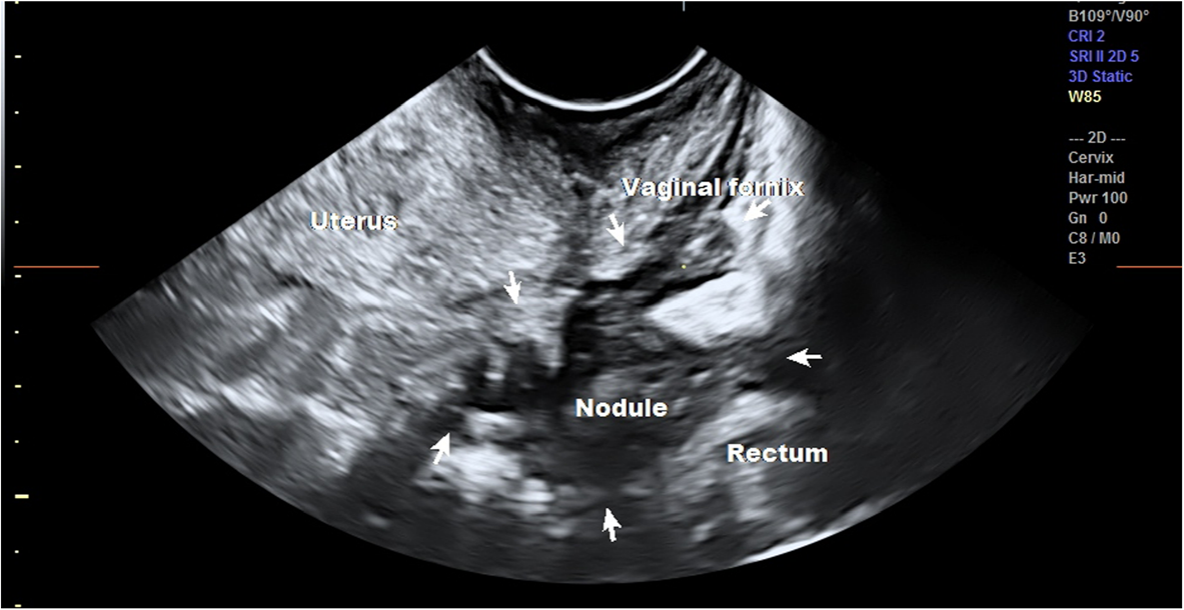
TVUS of a large vaginal nodule extending to the rectosigmoid. The sonographer interpreted this lesion as a rectosigmoid bowel lesion while the surgeon described it as a vaginal lesion. Arrows show extent of lesion

Thirty-nine women had vaginal nodules located in the vaginal fornix or rectovaginal septum. There was one false positive diagnosis by the sonographer and three false negatives. The false negatives were found to be lesions in the border between the rectovaginal and recto sigmoid area, which the surgeon described as rectovaginal lesions but which the sonographer classified as recto sigmoid bowel lesions. In all of these cases, the main symptom was infertility rather than posterior compartment complaints. The adhesions were so severe that the lesions were left in place in order to prevent causing extensive damage since there were no associated symptoms. The sonographer diagnosed the lesions accurately in 96% of the patients. Based on the LU-CUSUM curve, it takes 32 examinations to become proficient at diagnosing vaginal lesions (Fig. 3).

#### Pouch of Douglas obliteration

Forty-eight (51.1%) of women had pouch of Douglas obliteration. The graphs for vaginal nodules and pouch of Douglas obliteration are very similar. There were three false negative diagnoses, which were the same cases described above. There were four false positives by the sonographer. Based on the LU-CUSUM curve, it takes the sonographer 31 examinations to become proficient at diagnosing pouch of Douglas obliteration correctly.

#### Bowel lesions

**Fig. 4:**
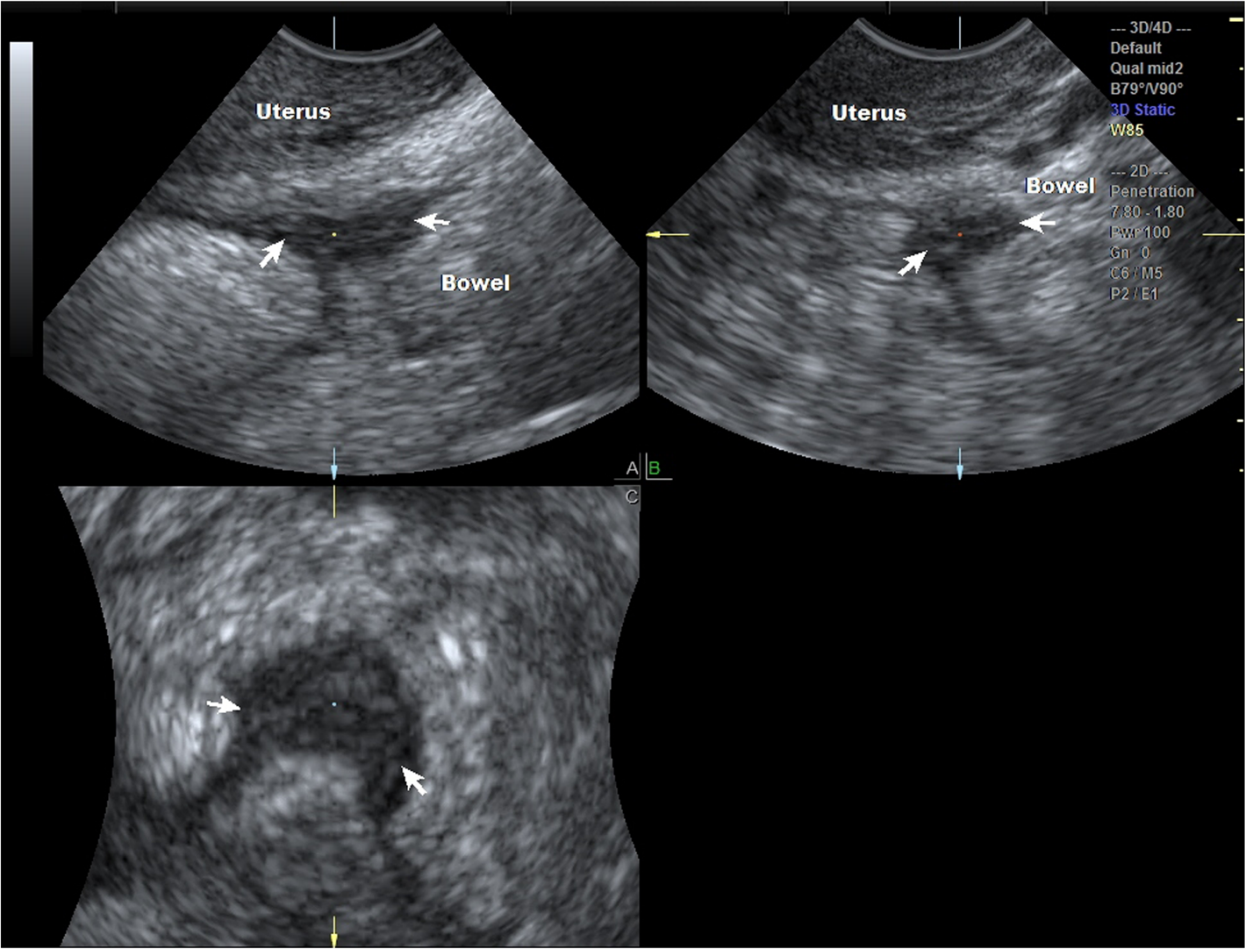
Multiplanar 3D image of TVUS of a bowel nodule behind the cervix. Nodule is shown in arrows

There were 20 lesions located in the rectum, sigmoid colon or higher pouch of Douglas. Four high lesions were missed by the sonographer, and these were located diffusely in the bowel far from the pouch of Douglas and were not accessed by the transducer. There was one false positive diagnosis by the sonographer early on in the learning procedure in case four. Based on the LU-CUSUM, curve it takes the sonographer 38 examinations to become proficient at diagnosing bowel lesions correctly (Fig. 4).

#### Uterosacral ligaments

Fifty patients had lesions in at least one of the uterosacral ligaments, which the sonographer diagnosed correctly in 65% of the patients. There were 20 false negative diagnosis by the sonographer were the lesions were deep in adhesions so that the uterosacral ligament was poorly visualized. Not all were resectable and were thus determined based on visualization during surgery. There were 13 false positives where lesions were assumed to occur in large conglomerates of adhesions but were not found after the adhesions were dissolved. In both of these situations, this did not affect the actual surgical procedure which was determined by the presence of vaginal, bowel nodules or pouch of Douglas obliteration. Based on the LU-CUSUM curve, it takes the sonographer 44 examinations to become proficient at diagnosing uterosacral ligament lesions correctly.

## Discussion

TVUS is increasingly utilized in the many developing subspecialties of gynecological imaging. Some of these fields entail specialized and structured training programs. Ultrasound diagnosis of endometriosis is such a developing field and is now considered to be the first-line imaging modality for disease mapping [9]. Despite this, it is not widely known up to this time how many clinical examinations are required in order to train an experienced ultrasonographer to be proficient in endometriosis imaging. This study shows that a sonographer trained in general gynecological ultrasound, who devotes time to learn TVUS for DIE mapping, can apparently achieve proficiency for diagnosing the varied types of endometriotic lesions within 44 examinations provided that the patient subsequently undergoes surgery and that feedback is available.

Learning a new procedure carries the risk of unacceptable standards, therefore continuous supervision and monitoring is required until an acceptable level of performance is reached. Several factors may influence the learning process, these being the procedure, the trainee, the mentor, the setting, all of which will affect the time and number of procedures required to complete the learning process [17].

Tammaa et al. [8] recently described the length of time required to become proficient in diagnosing pouch of Douglas obliteration and deep infiltrating endometriosis of the rectum with TVUS. However, their study defined the expert sonographer as the reference standard rather than laparoscopic surgery [8]. Bazot et al. [4] described their results of the learning curve of four inexperienced trainees in the diagnosis of endometriomas, finding LC-CUSUM to be adequate for this purpose, but their reference standard was also the expert sonographer rather than surgery [4]. Saba et al. [27] described the learning curve in the detection of ovarian and deep endometriosis by using MRI findings and by comparing them with surgical results. In this retrospective study, datasets were analyzed by the same performer before surgery and re-analyzed 12 and 24 months later to determine proficiency and results were compared between the analyses. They found that the performer’s expertise over time increased diagnostic accuracy, but that it takes at least 1 year of intensive training and at least 100 exams of patients with endometriosis, in order to acquire adequate experience. Alcazar et al. [28] evaluated an intensive training program for ultrasound diagnosis of adnexal masses using LC-CUSUM graph analyses. They found that this methodology can be used in the evaluation of the feasibility of a training program [28].

Recently, Piessens et al. [29] described their experience with a sonographer learning endometriosis screening in just 1 week of specialized training. Using the LU-CUSUM, this sonographer achieved competency within 38 scans for POD obliteration and 36 scans for bowel lesions. The study was also performed by an experienced gynecological sonographer, who had not previously performed DIE mapping. Overall, more than 100 examinations were performed [29]. Our findings also lend support to their findings, while we additionally described uterosacral ligament evaluation.

Our study has several strengths and limitations which should be acknowledged: a significant strength is the use of a validated statistical model for assessing the learning curve, the LU-CUSUM, which was described [17]. However, the acceptable and unacceptable failures were chosen arbitrarily based on previous reports. These values can be modified according to recommendations which are applicable to the specified procedure [17].

A significant strength of the present study is the fact that the analysis was performed against the reference standard of surgery with histo-pathological confirmation. The definitive diagnosis was only reached after surgery, which occasionally took place months after the ultrasound exam. The performance of these recommended 44 exams, with adequate surgical feedback, can take time depending on how many endometriosis surgeries are performed each week. In our study, this took well over a year and approximately 250 scans overall.

The accuracy of TVUS in diagnosis of endometriomas and DIE in our study is in concordance with previous reports [12–14] and with two recent meta-analyses [25, 26]. This provides further confirmation that TVUS remains the first-line imaging technique for suspected DIE. A sonographer with general gynecological experience is expected to diagnose all ovarian endometriomas correctly, as indeed was the case in our study. The present study also had a very good sensitivity for diagnosing bladder nodules, which were identified with the standardized approach. The preoperative diagnosis of bowel involvement and pouch of Douglas obliteration greatly impacts decision-making [9]. While competency is achieved reasonably fast, any missed diagnosis may be harmful. It is therefore imperative to ensure that the trainee and the surgeon speak the same language, as most inaccuracies may be the result of a different terminology. The surgeon was not blinded to the ultrasound report in this study, because he was expected to plan surgery based upon it. Furthermore, despite the high proficiency of our surgeons, some very deep lesions may not have been seen on laparoscopy and could have been missed [7]. The poorer performance which was obtained in the diagnosis of uterosacral ligament lesions is also in concordance with previous reports, and is in fact somewhat better in our study.

An additional limitation of our study is that all of the patients had TVUS because of suspected DIE, which may have caused a diagnostic bias. The analysis was performed in a tertiary referral center with a high number of advanced cases of severe endometriosis with multiple lesions, which does not reflect the standard patient population. Therefore, we analyzed more locations of endometriosis in an attempt to overcome this potential bias.

All of the TVUS examinations were performed by a single operator with training in general gynecological sonography, who had previously not been exposed to DIE scanning. This limits the generalization of our data to other potential operators, with a different degree of expertise in general gynecological imaging, who may in practice have a different learning curve. At the time of the study, this was the only trained sonographer at our center dedicated to endometriosis mapping, thus inter-observer comparisons cannot be discussed. Furthermore, the sonographer in question received tertiary referrals from other centers where eventually the surgery was undertaken. This may have affected the learning curve as well. Similar studies are necessary in order to learn more about the learning curve for different performance and competency levels. A trainee should ideally show evidence of satisfactory performance, before he or she can be encouraged to perform the procedure without supervision [17].

While the LC-CUSUM has been used here to assess the performance of a single individual, it may prove useful for monitoring the introduction of a new procedure in any setting where feedback is available and corrective actions can be implemented, or in monitoring new trainees, who have the advantage of being trained by an experienced DIE sonographer. It may also prove useful for professional societies that are responsible for developing guidelines for good practice [17]. It is clear that any general gynecologic sonographer could benefit from such training, provided that feedback on diagnosis is available. We believe that our experience may serve as a guiding point to other operators who aim to learn endometriosis mapping. With the new consensus opinion recently published by Guerriero et al. [30], better standardization and education is becoming feasible.

## Conclusions

This study shows that a sonographer trained in general gynecologic ultrasonography, who devotes time to learn TVUS for DIE mapping, can achieve proficiency for diagnosing the major types of endometriotic lesions after examining less than 50 patients who subsequently undergo surgery in a training setting. This goal can be accomplished within a reasonable time frame in many tertiary referral centers involved in the care of endometriosis patients. Diagnostic accuracy may be further advanced in these centers by improving agreement on terminology and feedback between surgeons and imaging specialists. Determining the learning curve for DIE TVUS mapping can aid training programs in dedicated endometriosis centers and may be of value to general gynecology TVUS education programs as well. Improving training for new ultrasound performers may in the future enable earlier non-invasive diagnosis of endometriosis which will have a favorable impact on the healthcare of women. Despite the fact that we have described the experience of a single operator, we believe that this may encourage other gynecological ultrasound experts to further their expertise into the field of endometriosis mapping.

## Appendix

The TVUS were carried out using a transvaginal 5–9 MHz probe with 2D/3D capabilities (Voluson 730 or E6, GE Medical Systems). Images were interpreted in real-time and were stored for later analysis. The examination was performed in a standardized way for all patients, which included a thorough evaluation of all pelvic viscera, in search of endometriosis findings. The examination was performed at any time of the menstrual cycle regardless of hormonal therapy. Bowel preparation was not utilized and is not standard practice at our center. The probe covering is filled generously with ultrasound gel to act as acoustic window. The uterus was studied in a midsagittal plane identifying the uterine cavity and cervical canal, moving to the right and left in order to encompass the entire uterine cavity. The probe was then rotated 90 ° to the left to view the uterus in the transverse plane. The myometrium was thoroughly evaluated for any abnormalities in all planes. The operator then evaluated the adnexae for lesions consistent with endometriomas, tubal adhesions, DIE, pouch of Douglas obliteration, the parametria, recto sigmoid, vagina and retro cervical area, and organ movement.

On B-mode ultrasound, endometriomas were defined as uni- or multilocular cysts with ground glass echogenicity of the cyst fluid and no papillation with detectable blood flow [18]. When the B-mode was inconclusive, power Doppler imaging was performed to exclude corpus luteum cysts [19]. The location (bilateral, right, or left) and size of each endometrioma were evaluated. In the presence of multifocal or bilateral endometriomas, the largest endometrial cyst was taken into account and measured.

The peritoneal surfaces of the anterior compartment were visualized. Bladder nodules were seen as hypo-echoic lesions in the vesicouterine pouch or bladder wall, with or without cystic areas and regular or irregular margins, bulging towards the lumen. The distal ureters were also seen. Bladder adhesions of the vesicouterine pouch were evaluated by the presence or absence of movement between the uterus and the bladder. Infiltration of the bladder wall was noted [20].

Posterior compartment lesions were evaluated in the vagina, posterior and lateral vaginal fornices, the retro cervical area with torus uterinum, the parametria laterally, the rectovaginal septum, and bowel. Vaginal nodules were visualized as hypo-echoic lesions in the vaginal fornix. Bowel involvement was visualized as a hypo-echoic lesion adhering to the bowel wall and bowel layers were examined for penetration. The number of lesions, their size in three dimensions and location from the anus were described. Uterosacral ligaments involvement was searched for in the parametria in the paracervical area and was seen as an infiltrating irregular hypoechogenic tissue [20]. When there were multiple adhesions in the area of the uterosacral ligaments, they were interpreted as involved with DIE. Organ mobility was evaluated in the anterior and posterior compartment. Movement of the posterior surface of the uterus, cervix, or vagina in relation to the bowel was examined in order to determine pouch of Douglas obliteration, previously described as the sliding sign [21, 22].
